# Assessments of Acoustic Environments by Emotions – The Application of Emotion Theory in Soundscape

**DOI:** 10.3389/fpsyg.2020.573041

**Published:** 2020-11-20

**Authors:** André Fiebig, Pamela Jordan, Cleopatra Christina Moshona

**Affiliations:** ^1^Engineering Acoustics, Institute of Fluid Dynamics and Technical Acoustics, Technische Universität Berlin, Berlin, Germany; ^2^Amsterdam Centre for Ancient Studies and Archaeology, University of Amsterdam, Amsterdam, Netherlands

**Keywords:** soundscape, emotion, mood, appraisal, soundscape descriptors, affect, affective quality

## Abstract

Human beings respond to their immediate environments in a variety of ways, with emotion playing a cardinal role. In evolutionary theories, emotions are thought to prepare an organism for action. The interplay of acoustic environments, emotions, and evolutionary needs are currently subject to discussion in soundscape research. Universal definitions of emotion and its nature are currently missing, but there seems to be a fundamental consensus that emotions are internal, evanescent, mostly conscious, relational, manifest in different forms, and serve a purpose. Research in this area is expanding, particularly in regards to the context-related, affective, and emotional processing of environmental stimuli. A number of studies present ways to determine the nature of emotions elicited by a soundscape and to measure these reliably. Yet the crucial question—which basic and complex emotions are triggered and how they relate to affective appraisal—has still not been conclusively answered. To help frame research on this topic, an overview of the theoretical background is presented that applies emotion theory to soundscape. Two latent fundamental dimensions are often found at the center of theoretical concepts of emotion: valence and arousal. These established universal dimensions can also be applied in the context of emotions that are elicited by soundscapes. Another, and perhaps more familiar, parallel is found between emotion and music. However, acoustic environments are more subtle than musical arrangements, rarely applying the compositional and artistic considerations frequently used in music. That said, the measurement of emotion in the context of soundscape studies is only of additional value if some fundamental inquiries are sufficiently answered: To what extent does the reporting act itself alter emotional responses? Are all important affective qualities consciously accessible and directly measurable by self-reports? How can emotion related to the environment be separated from affective predisposition? By means of a conceptual analysis of relevant soundscape publications, the consensus and conflicts on these fundamental questions in the light of soundscape theory are highlighted and needed research actions are framed. The overview closes with a proposed modification to an existing, standardized framework to include the meaning of emotion in the design of soundscapes.

## Introduction

The field of soundscape focuses on how people experience their surrounding acoustic environments. This disciplinary position stands in contrast to the field of noise control, which focuses on human response to loudness and annoyance derived from environmental noise exposure. Soundscape’s broader view of sonic experience naturally points to the potential of incorporating findings from affect, emotion and appraisal research, particularly as both noise and soundscape fields already borrow related language and concepts (e.g., annoyance as a metric). Human responses to the (acoustic) environment may even be a reflection of evolved motivational and affective systems, promoting survival through preferences for certain environments and avoidance of others (van den Bosch et al., 2018). In order to place potential benefits stemming from emotion theory within the context of soundscape research and assessment, a brief review of emotion theory is first necessary.

### Emotion Theory and Research

Emotions are a nearly constant aspect of the human phenomenal experience (Nielsen and Kaszniak, 2007), with states such as fear, happiness, boredom or amusement arising without conscious effort. With a subject–and lived experience–so familiar to everyone, the scientific approach to the study of affective and emotional states^1^ faces a challenge: any emotion theory must stand up to scientific rigor alongside any individual’s common-sense examination. This dual standard for research on emotion is likely one reason why an established theory of emotion does not yet exist (Müller and Reisenzein, 2013).

Even so, research abounds. Rottenberg et al. (2007) have traced the explosive growth of research during the past few decades, leading to new theories, methods, and findings. Coan and Allen (2007) substantiate this, highlighting the great diversity of methodological approaches that are currently driving emotion science. Many researchers address the issue of separating emotion from cognition, the relation of cause and effect, the distinction between basic and complex emotions, conscious and unconscious aspects of emotions, the relation between rationality and emotion, and the true origin of emotion. Some key texts along these lines will be highlighted in the discussion that follows. Overall, emotions seem to be an integral concept that subsumes psychological stress and coping, uniting motivation, cognition and adaptation in a complex configuration (Lazarus, 1991).

As such, emotion is difficult to tackle by a single traditional psychological theory. Yet the study of the nature and structure of emotion has a long tradition that is still developing. It was recognized in the 19th century, the early days of psychophysics as a field, that body and mind are deeply intertwined. James had concluded that, if we consider a strong emotion and try to partition the feelings of its characteristic bodily symptoms from our consciousness of the emotion, there remains no “mind-stuff” from which the emotion can be constituted (James, 1884). Over 100 years later, Gross acknowledged that this tension remains rather unresolved: the “*definition of the construct emotion is* [still] *in a state of conceptual and definitional chaos and remains a heavily freighted term full of imprecision*” (Gross, 2010). Therefore, deriving a definition of emotion remains “[…] *a difficult matter [*and*] a definition of emotion can only be a product of theory*” (Frijda, 1986). Furthermore, the attempts by different disciplines to access emotion research via their own concepts and methods seems to impede the development of a universal view on emotion (Müller and Reisenzein, 2013). But Ekman has pointed out that what is really needed–rather than a comprehensive, universal theory of emotion–is to have a separate theory for each emotion in order to capture its unique aspects (Ekman, 1994).

There are a few aspects of emotion that appear to be recognized across disciplinary borders, which will be addressed in more detail:

• Emotions are internal, mostly conscious, and relational.
• Emotion can manifest in different forms. Frequently, the emotion phenomena are differentiated according to physiological responses, experiences, and behavior.
• Emotions are short-lived phenomena and must be distinguished from mood and attitude by means of duration. As emotions are short-lived processes affected by the moment, mood and attitude are more stable, less affected by the moment, and long-lasting.
• Emotions serve a purpose.

#### Relational Aspects

Emotions are internal psychological experiences, yet they are both relational and elicited by others or a specific encounter with an environment (Lazarus, 1991). The experience of an emotion can usually be linked to a specific, defining moment and triggered by a specific object, which makes emotions different from mood and attitude (Gray and Watson, 2007). The events that elicit emotions also appear to fulfill a special role – they are not simply stimuli. In fact, they appear to act through their significance, their meaning, their rewarding or aversive nature (Frijda, 1986).

As mentioned, the primary function of emotions is to provide feedback for reacting effectively to the environment (Clore, 1994). The processes of appraisal can be consciously controlled in part, but elements of the appraisal process, such as basic emotions (e.g., happiness, sadness, anger, and fear) and their functions, remain closed to comprehensive cognitive penetration (Frijda, 1986). The magnitude of emotional response an individual experiences is strongly related to the magnitude of emotional stimulus, and in this sense emotion is relational. But individuals experience emotions differently, attributable to a person’s inherent emotional predispositions, what Larsen and Diener call personal emotionality (Larsen and Diener, 1987). Thus, the element of stimulus is always an intrinsic property that affects a human’s emotions. At the same time, knowledge about a stimulus’ significance to well-being, inherent in the concept of appraisal, contributes to one’s personal meaning that also drives emotional responses (Lazarus and Smith, 1988).

Although it is widely assumed that humans have access to their emotions and can report on them (cf. Müller and Reisenzein, 2013), it is likely that they have no direct access to the causal connections between external forces and internal responses. Individuals are simply limited in their ability to track the complex causal story of their emotions (Russell, 2003); even though emotions themselves are conscious, any appraisals leading to them are often unconscious (Clore, 1994). Sometimes the cause is obvious, but at other times individuals experience a change in affect without exactly knowing why (Russell, 2003). While understanding the stimulus and context for a response is an intrinsic feature of soundscape work, the difficulty in identifying the causes of emotions presents a challenge for emotion theory and soundscape research alike.

#### Forms

Emotions can be triggered by all human sensory systems, demonstrating an intrinsic link between emotional and physiological responses (Hume and Ahtamad, 2013). When looking at affective pictures, patterns of physiological change are found that vary with reports of affective valence and arousal (Bradley and Lang, 2000). Similar patterns of physiological reactions are elicited by affective pictures (Lang et al., 1993), affective sounds (Choi et al., 2015) and films (Fredrickson and Kahneman, 1993). In another illustrative study, when individuals viewed unpleasant pictures, a cardiac deceleration, a large skin conductance response, observable increases in corrugator (frown) electromyogram (EMG), a larger scalp-recorded positivity, and a potentiation of the startle reflex were observed (Gray and Watson, 2007). As emotions manifest in varying forms with many sub-components (Juslin, 2013a), the emotional response can be observed and measured in different ways, such as affective reports, physiological reactivity, and overt behavioral acts (Bradley and Lang, 1994). Distinguishing the various forms of emotional response and developing research methodologies to measure them is an important consideration for formulating appropriate soundscape studies.

#### Duration

**FIGURE 1:**
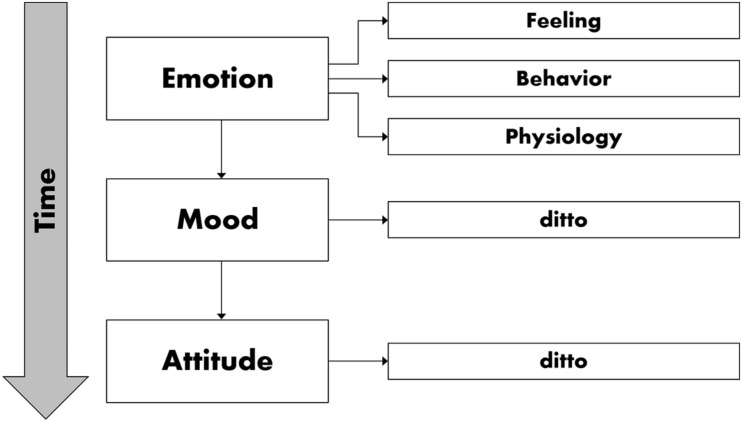
Simplified model of emotion adapted from Gross (Gross, 2010).

In contrast to longer-lived moods, which can last hours or even days, emotions are intensive yet brief (Gray and Watson, 2007). Emotions and mood can be linked by duration in some circumstances, such as when a series of mild positive events together result in a positive mood over time (Davidson, 1994). So moods and emotions can be seen to interact dynamically (though the duration criterion only applies to a limited extent). As moods last longer, their causes are more remote in time and less salient compared to emotions, which are closer to the cause and thus seem to be more conscious (Clore, 1994). Figure 1 illustrates the role of time in distinguishing emotion, mood, and attitude, distinctions that are especially relevant in soundscape studies. Moreover, the phenomenon of *duration neglect* is frequently discussed. This term refers to the insignificance of duration for reporting summarized affect of longer periods. The irrelevance of duration was observed in several empirical contexts, like pain or loudness perception or the displeasure of movie clips, and is important for reporting about emotions as well.

#### Purpose

By means of elicited emotions, humans can rapidly recognize and quickly adapt necessary behavioral responses. Emotion thus can be understood as a driver of behavior. Emotions most often arise in situations where adaptive action is required (Davidson, 1994); they provide a means for dealing with fundamental situations quickly without much elaborate planning (Ekman, 1992). This could help explain the observation that emotional stimuli are prioritized in perception, are detected more rapidly, and gain access to conscious awareness more easily than non-emotional stimuli (Brosch et al., 2010). Such dynamics are reflected in the ability of humans to detect even subtle emotional nuances in speech and to adapt to them accordingly, for instance (Paeschke, 2003). Going further, Lang et al. (1993) state that *valence* and *arousal* represent primitive motivational parameters that define a general disposition to approach or avoid stimulation. Because judgments of pleasure and arousal reflect (in part) this motivational imperative, Lang et al. (1993) postulate a correlation between brain state and evaluation. Many researchers addressing emotion theories agree that emotional stimuli and emotional responses represent a special type of input – they both represent high relevance for survival and well-being by preparing the organism for action or decision (Gray and Watson, 2007; Brosch et al., 2010). Emotions inform the individual of the nature and importance of events, and the magnitude of feelings motivates an individual to focus quickly on relevant considerations (Clore, 1994). Lang et al. (1993) have proposed that the multi-dimensional emotional experience underlying affective judgments represents a bi-motivational structure involving two systems of appetitive and defensive motivation in life. However, Hall et al. (2013) believe that the emotional experience underlying real environments is perhaps too complex to be captured by only two motivational factors. This framing supports the idea that a number of physiological systems are primarily sensitive to emotional activation across sensory modalities rather than to a specific mode of presentation of stimuli, such as images or sounds (Bradley and Lang, 2000).

Though a consolidated theory of emotion is the subject of ongoing research, the specific role of emotion for managing inner and outer worlds, including characteristics and features, is well acknowledged and bears significance for soundscape research.

### Introduction of Soundscape

The idea of soundscape was introduced in the late sixties as a contrast to the conventional perspectives of noise control and environmental policies at that time. According to Schafer (2012) (one of the founders of soundscape) all urban sounds should be the subject of study, “not merely those that were unpleasant or dangerous.” This position significantly broadened the view on the distinct effects of sound on humans beyond the environmental noise abatement paradigm, which considered noise solely as a waste and the least annoying acoustic environment to be one free of any (unwanted) noise. Today it is known that the mere reduction of noise levels does not necessarily lead to more positive appraisals of an environment (van den Bosch et al., 2018).

The widening of scope to include both positive and negative sonic effects has led to a research shift from physical stimulus alone to human auditory sensation and its interpretation. The first concepts of soundscape emphasized that an acoustic environment is understood by those living within it and creating it (Truax, 1984). This early notion of soundscape was echoed in the recent international standard on soundscape, ISO 12913-1: “*Soundscape is an acoustic environment as perceived or experienced and/or understood by a person or people, in context*” (International Organization for Standardization, 2014). The recognized term soundscape thus refers to the perceived acoustic environment of a place, whose character is the result of the action and interaction of natural and/or human factors (Kang et al., 2016). Soundscape research focuses on perception under contextual conditions.

Research on soundscape has become more and more popular, and the field continues to explore new facets of how acoustic environments affect human perception. The overarching aim of soundscape research is to understand the relationship of people and their acoustic environment, examining the sounds that people value or oppose as well as the shifts in reaction due to changing location and activity (Kamp et al., 2016). For that purpose, various approaches have been proposed for studying the meaning of (environmental) sounds for humans and for determining the specific characteristics of perception; one of the most important and relevant for soundscape study is the verbal report.

## Verbal Reports to Study How Humans Emotionally React to Environments

In the late nineteenth century, Wundt recognized that emotions are composed of three major dimensions — “*Lust*” and “*Unlust*” (pleasure and displeasure), “*Erregung*” and “*Beruhigung*” (excitement and tranquilization), and “*Spannung*” and “*Lösung*” (tension and relaxation) (Wundt, 1906), terms which still seem current. Many psychologists since Wundt have agreed that the dimensional concept of emotion is a useful approach to provide a taxonomy of emotions and have searched for broadly applicable generic labels (Gehm and Scherer, 1988). Dimensional verbal reports of this variety would be also familiar to recent soundscape researchers (Axelsson et al., 2010) and will be addressed later on.

However, there continues to be a lively debate about the fundamental dimensions that characterize the phenomenal space of emotion experience (Nielsen and Kaszniak, 2007). Many researchers have followed the dimensional theory approach in the belief that affect and emotion are composed of a small number of general dimensions that are usually thought to be independent of each other. Gray and Watson (2007) pointed out that “researchers began to adopt models that bypassed these discrete affects and posited few underlying dimensions.” As discussed in the introduction, emotions present a complex mixture of consciously accessible and intuitive responses that are captured in dimensional models. Although emotions have both behavioral and physiological characteristics, Lazarus (1991) concluded that emotions are above all psychologica+l. Clore (1994) emphasizes that “[…] *one cannot have an unconscious emotion, because emotion involves an experience, and one cannot have an experience that is not experienced*.” As psychological states that are consciously accessible by their receivers, emotions can thus be effectively studied using participatory self-report methods. Intriguingly, such assessments appear to be stable over time: considering retrospective reporting of emotions over specific time intervals, it seems that participants have little trouble giving relatively reliable and valid emotion ratings (Robinson and Clore, 2002).

Continuing the explorations of dimensional models, Osgood et al. (1975) observed fundamental semantic dimensions such as *evaluation*, *activity* and *potency* by investigating the nature of meaning of languages using the semantic differential method. The dimensions across later research using the semantic differential method frequently bear a striking resemblance to the dimensions observed by Osgood et al. (1975) — *hedonic valence*, *activity* and *potency* (Gehm and Scherer, 1988). An influential work in line with Osgood et al. (1975) was later published by Mehrabian and Russell (1974) using the multivariate research on affective language, finding that the principal variance in emotional meaning appears to be sufficiently explained by a limited set of basic emotional responses to all situations: the main independent factors *pleasure, arousal*, and *dominance*. *Pleasure* must be distinguished from preference or liking, while *arousal* describes a single dimension ranging from sleepy to excitement. However, less attention is paid to dominance in research and models are used with only two axes: the degree of pleasure oriented horizontally, and the degree of arousal oriented vertically (Bakker et al., 2014). These terms have recently been adopted within soundscape, so their application in emotion research bears a moment of further consideration.

The dimensions identified as *pleasure* and *arousal* are frequently obtained in factor analytic solutions based on a set of data consisting of a heterogeneous sample of adjective items and a set of rated stimuli. Factors that emerge are expected to denote fundamental affective or perceptual components. Russell et al. (1981), building on the work from Mehrabian and Russell, developed a circumplex model of affective states elicited by environments, a circle in a two-dimensional bipolar space based on the dimensions of *pleasure-displeasure* and *arousal-sleep*. In a circumplex model, descriptors are systematically arranged around the perimeter of a circle leading to bipolar dimensions, revealing the relationships between two separate dimensional scales. Bakker et al. (2014) refer to the underlying mechanism to explain *pleasure* and *arousal* as related to the *degree of order* and *variation*.

The two-dimensional model has received extensive empirical support as the same basic two-dimensional structure consistently emerged in self-report data (Gray and Watson, 2007). A similar, though not identical, model receiving attention was proposed by Watson and Tellegen, who emphasized the importance of *negative affect* and *positive affect* as independent dimensions. The *negative affect* reflects unpleasant affective states with low or high arousal states, whereas the *positive affect* dimension ranges from enthusiastic and excited to sleepy and drowsy (Watson and Tellegen, 1985). There are some debates surrounding the bipolarity and independence of dimensions implied in the different models [e.g., Is positive affect the bipolar opposite of, or is it independent of, negative affect? (Feldmann Barrett and Russell, 1998)]. Yet Russell and Carroll (1999) detected no substantive controversy and a consensus on a descriptive structure of current affect seems imminent (Feldmann Barrett and Russell, 1998). Although meaning attributed to environments contains both affective and perceptual-cognitive components with the two highly interrelated, the detected latent fundamental dimensions focus specifically on emotions (Russel and Pratt, 1980). The identified dimensions of affective qualities are currently applied by numerous researchers, though there is and will be a continuing debate about the interpretation of the dimensions with their underlying mechanisms (Bakker et al., 2014). Russell himself acknowledged that his own dimensional model of emotion fails to provide a sufficiently rich account of *prototypical emotional episodes* such as distinguishing between *fear* or *anger* (Russell, 2003), fuelling the debate about the dimensional or categorical nature of emotions. However, the extensive evidence from similarity judgments between emotion related adjectives, judgments of facially expressed emotions, self-reported mood, and psychophysiological measurements indicates that two dimensions are usually considered to be sufficient (Västfjäll et al., 2002). The same basic two-dimensional structure consistently emerges in self-report data, leading to the conclusion that this structure is considered fundamental or *basic* as described by Watson et al. (1999).

As verbal reports frequently refer to a certain period experienced in the past, aspects of duration that were discussed in the introduction might become relevant. Delayed judgments of a past episode reduce the relevance of the episode’s total duration, salient single moments become even more important, and at the same time other distinct emotions are glossed over during the episode (Fredrickson and Kahneman, 1993). Emotion reporting often requires participants to remember and summarize their experiences when giving an account of past emotions. Retrospective biases, such as recollection and weighing of specific moments of an experience or belief-based reconstruction, must be considered in such reporting (Robinson and Clore, 2002). It is very likely that, if retrospective measures of emotion experiences are requested, respondents create emotion reports using different types of processing strategies – retrieval of prior experiences versus reconstruction of the past experiences, for instance (Feldmann Barrett, 1997). Altogether, it appears that two distinct emotional selves are available: one that lives in the moment and one that lives in the abstract, which means that distinct sources of self-knowledge are accessed under different reporting conditions when referring to ongoing or to retrospective emotions (Robinson and Clore, 2002). According to Gärling et al. (2020), in the context of emotional wellbeing, the most valid and reliable method is the self-report on momentary states (e.g., *How do you right feel now?*), because instantaneous self-report measures are barely influenced by memory distortions and subject of meta-analyses. There is still a significant lack of understanding in the role of duration, memory, and integration heuristics on environmental sound-induced emotion and its reporting; systematic investigations on these issues are rarely conducted. However, the general value of self-reports for emotion research cannot be questioned and prove essential for soundscape research as well.

## Emotions and Their Dimensions in Soundscape Research

Soundscape research generally acknowledges that the process of perceiving and assessing environmental sound is multi-dimensional and the simplifying concept of annoyance is insufficient for thorough analysis (Schulte-Fortkamp and Fiebig, 2016; Jordan, 2019). Therefore, the consideration of basic and complex emotions within soundscape work is logical, and emotion theory is increasingly gaining significance in applied soundscape research. Sounds have been demonstrated to elicit emotional processes in experimentally controlled laboratory contexts with standardized affective stimulus databases, i.e., the International Affective Digitized Sounds IADS-I (Bradley and Lang, 1999) and IADS-II (Bradley and Lang, 2007b). It seems that environmental sounds carry biologically significant information reflected in human emotional responses, and that emotions work to optimize adaptive responses to biologically meaningful events (Ma and Thompson, 2015). However, research on the auditory system has been less intensively performed in the past than research on the visual system (Yang et al., 2018).

### Emotion in Music Versus Soundscape

One sound-related area that has not been neglected by emotion is music. It seems beyond question that music as an auditory event can provoke emotions. According to Juslin and Västfjäll (2008), emotions can be evoked in different ways and to different degrees by different stimuli, and music is no exception. The dominant approaches to conceptualize emotions are classified as *categorical* and *dimensional*. As such, Juslin (2013b) concluded based on empirical evidence that musical expression of emotion is likely to involve emotion categories rather than mere dimensions. The ability of music to affect human emotions is derived from its arrangement and not just from its sonic material. Music seems to channel the complexity of the acoustic world into an ordered form (Truax, 1984). This suggests that acoustic environments are capable of evoking a similar set of emotions. In one study, changes in acoustic attributes that evoke emotional responses in speech and music (e.g., frequency spectrum, intensity, and rate) were observed also to induce emotions when perceived in environmental sounds (Ma and Thompson, 2015). According to Ma and Thompson (2015), this observation aligns with the *musical protolanguage hypothesis* that speech and music originated from a common emotional signal system based on the imitation and modification of sounds in the environment. Truax (2016) has observed that, although intense affective responses as expressions of emotions through speech and music have been studied extensively, the equivalent role of environmental sounds has unfortunately so far been ignored.

Despite the fact that the concept of ‘soundscape’ is originally rooted in music (Kang et al., 2016), as well as Schafer’s assertion that “from art, particularly music, we will learn how man creates ideal soundscapes” (Schafer, 1977), the mechanisms connecting music and emotions are substantially different to the mechanisms at work in soundscape-elicited emotions. Music is (almost always) composed intentionally to arouse a wide range of emotions. Listeners usually consciously experience music, engage in decoding “intended” emotions and are aware of the manifold stylistic elements to “inspire” the audience. The effect of acoustic environments on emotions is more subtle and often goes unnoticed. Acoustic environments are rarely explicitly ordered or designed to induce emotions. Accordingly, Ma and Thompson observed that core acoustic attributes relevant for elicited emotions by music and speech are also relevant for the emotional character of environmental sounds, but the authors simultaneously explain that acoustic environments have other acoustic attributes with emotional significance (Ma and Thompson, 2015). It is evident that the findings of emotion theory regarding music cannot be directly mapped onto soundscape contexts.

### Dimensional Models in Soundscape

Soundscape researchers searching for basic soundscape-related emotions and their underlying indicators have strongly based their concepts on common findings in environmental psychology with respect to the *dimensional* notion of emotion and affect. For example, Russell et al. (1981) explained that “[…] *exciting places are both pleasant and arousing. Peaceful and comfortable places are also pleasant but unarousing. Frightening and harsh places are unpleasant and high in arousing quality. Depressing places are unpleasant and unarousing.*” (Russell et al., 1981). These observations pave the way for similar understandings of the effects of acoustic environments on people. Bradley and Lang (2000) discovered that physiological responses elicited by visual stimuli appear to be organized fundamentally along dimensions of *pleasure* and *arousal*, implicating underlying motivational systems of appetite and defense and suggesting the likely intermodal generalizability of these dimensions. Consequently, Mehrabian and Russell (1974) believed in a common core of responses as an immediate result of stimulation to all types of stimuli regardless of the sense modality stimulated, a stance which has been influential for soundscape researchers looking for fundamental emotion dimensions elicited by acoustic environments.

Emotion theory holds that pleasant and unpleasant feelings form a bipolar continuum (Russell and Carroll, 1999), which dovetails with the fundamental soundscape concept that sound is a resource (Schafer, 1977). The soundscape approach focuses on sounds that are preferred by humans, as opposed to noise control’s focus on sounds of discomfort – those causing sleep disturbance, annoyance, communication interference, or effects on cognitive processes (Brown and Lam, 2015). As emotion theory centers on the relationship between person and environment rather than on either environment or intrapersonal events alone (Lazarus, 1991), the current trend in soundscape research to study emotions is propitious. Moreover, because emotions seem to have evolutionary roots in preparing the organism for action, the meaning of emotions, their link to the acoustic environment, and evolutionary needs are understandably subject to discussion. The circumplex concept as an approximation of fundamental emotions is a convenient and heuristic affect model in this case. It is not surprising that soundscape-related emotion researchers have adopted this notion of elicited emotions and that the affective concepts of Mehrabian and Russell attributed to environments frequently serve as a starting point.

**FIGURE 2:**
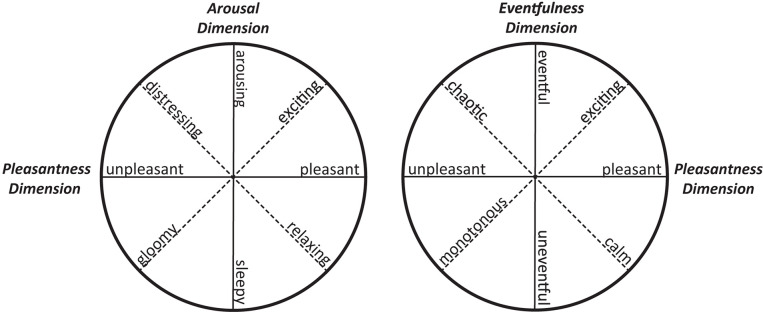
Two-dimensional representation of the affective quality attributed to physical environments generally (left, adapted from Russell et al., 1981) and to acoustic environments in particular (right, adapted from Axelsson et al., 2010).

Indeed, Bjork (1985) replicated Mehrabian and Russell’s dimensions *pleasantness* and *arousal* in the context of elicited emotions by natural sounds. Later, Axelsson et al. (2010) intensively studied the affective qualities attributed to acoustic environments and proposed a few basic dimensions of affective qualities for soundscapes that reflect the main features of the circumplex model (Russell et al., 1981). Figure 2 presents a side-by-side comparison of Russell’s research and Axelsson et al. (2010)’s recent application of the dimensional model in soundscape contexts:

In their work, Axelsson et al. (2010) discovered the basic dimensions *pleasantness*, *eventfulness* and *familiarity* in the context of soundscape. However, Axelsson et al. (2010) point out that the small variation in familiarity of soundscapes results means that the familiarity component is considered to be of limited importance for applied work, though it may at least be relevant to basic research. In an interesting convergence, the underlying dimensions of affect that were detected for acoustic stimuli are similar to those determined for affective image processing (Bradley and Lang, 2007a; Axelsson, 2011). The first two independent dimensions, *pleasantness* and *eventfulness*, might reflect evolutionary needs across sensory domains, promoting survival by preferring certain environments and avoiding others (van den Bosch et al., 2018). In 1984, Truax had already conjectured the main soundscape-related dimensions *variety* and *coherence*, which seem to be close to Axelsson’s proposed dimensions *pleasantness*, *eventfulness* and *familiarity*. *Eventfulness* can be considered as a semantic dimension of (auditory) *order* and *variation.* For example, a busy flea market with bustling activities or a popular, overcrowded urban city park are commonly perceived as eventful.

### The Diversity of Soundscape (Emotion) Dimensions

Beyond the typical dimensions related to *hedonic valence* and *arousal*, sometimes soundscape investigations explore other or additional dimensions through statistical analysis to reduce the number of observed variables to a few fundamental ones. The dimensions proposed as appropriate to soundscape have expanded significantly in recent years. Aletta et al. (2016) suggested *appropriateness* as a third soundscape dimension for consideration. An encountered situation is usually matched against existing cognitive schemes, i.e., personal expectations; thus *appropriateness*, the level of match between expectation and real-world situation, can influence an individual’s positive affective responses to a situation. In contrast, inappropriate matches lead to negative affective responses (van den Bosch et al., 2018), again harkening to survival origins. Tarlao et al. (2019) determined basic dimensions that they labeled *appreciation*, *dynamism*, and *monotony* as separate factors. Cain et al. (2013) and Davies et al. (2013) observed *calmness* and *vibrancy* as principal dimensions of emotional responses to soundscapes, which appear to be similar to the rotated circumplex model of Axelsson et al. (2010). Aletta and Kang (2018) investigated descriptors predicting *vibrancy* and surprisingly did not observe a significant correlation with pleasantness. This may indicate an independent dimension or, as the authors suggest, an accidental measurement of *eventfulness* being obtained through the research (Aletta and Kang, 2018). Andringa and van den Bosch (2013) referred to the main dimensions *pleasure* and *activation* in their work. Welch et al. (2019) observed the soundscape dimensions *calming*, *protecting*, *hectic*, *belonging* and *stability*. Yu et al. (2016) extracted the major factors of soundscape perception to be *preference*, *loudness*, *communication*, *playfulness*, and *richness* in the context of urban shopping streets. Sudarsono et al. (2019) derived the dimensions *privacy, disturbance*, *dynamic*, *fear*, and *satisfaction* in crowded third-class hospital wards. Zhang and Kang (2020) tried to distinguish between felt and perceived emotions induced by soundscapes and identified in their factor analysis dimensions labeled *comfort*, *enjoyment, excitement, desolation, tension*, or *familiarit*y indicating a mixture of *hedonic valence* and *activation* dimensions.

Table 1 lists the detected soundscape dimensions in selected publications. As shown in Table 1, most of the listed studies are based on controlled laboratory experiments. Field surveys were only rarely conducted to determine fundamental dimensions of emotions in soundscape.

**TABLE 1:** Soundscape descriptors as emotion dimensions.

Authors	Detected dimensions	Applied method
Truax, 1984	Coherence, variety**	Theoretical deduction
Bjork, 1985	Pleasantness*, arousal**	Semantic differential method, principal components analysis (L)
Västfjäll et al., 2003b	Valence*, activation**	Multiple rating scales, sum of scales (L)
Axelsson et al., 2010	Pleasantness*, eventfulness**, familiarity	Semantic differential method, principal components analysis (L)
Cain et al., 2013	Calmness*, vibrancy**	Semantic differential method,^2^ principal components analysis (L)
Andringa and van den Bosch, 2013	Pleasure*, activation**	Based on literature
Yu et al., 2016	Preference*, loudness, communication, playfulness richness**	Semantic differential method
International Organization for Standardization, 2019	Pleasantness*, eventfulness**	Defined based on literature
Tarlao et al., 2019	Appreciation*, dynamism**, monotony	Semantic differential method, principal components analysis (F)
Sudarsono et al., 2019	Privacy, disturbance*, dynamic**, fear, satisfaction*	Semantic differential method, principal components analysis (F)
Welch et al., 2019	Calming**, protecting*, hectic, belonging, stability	Qualitative method analyzing written text, semantic differential method, principal components analysis (L)
Zhang and Kang, 2020	Enjoyment*, excitement**, desolation, tension, familiarity (*related to felt emotions*)	Semantic differential method, factor analysis (L)
Zhang and Kang, 2020	Comfortable*, festive**, desolate, familiar, attractive*, nostalgic (*related to perceived emotions*)	Semantic differential method, factor analysis (L)

Dimensions are marked with asterisks if they resemble the Mehrabian and Russell’s pleasantness (*) and arousal (**) dimensions. The letters L (laboratory experiment) and F (field study) indicate the general type of the study. ^2^The reference to the “semantic differential method” in this table refers to the use of multiple category rating scales to be judged by participants that vary in format and design.

### Universality of Dimensions

For Jeon et al. (2018), the components *pleasantness* and *eventfulness* commonly identified in several studies from different countries appear to be universal across languages, cultures, and environments. The ISO/TS 12913-2 expresses the general appreciation for this model in proposing a questionnaire consisting of response scales related to different affective attributes (International Organization for Standardization, 2018). The use of multiple ratings across sets of scales in the circumplex allows for reliable assessments of core affects including main emotional dimensions as recommended in the ISO/TS 12913-2 and ISO/TS 12913-3.

However, although emotions are understood to be essentially universal, cultural differences in emotions are frequently reported, suggesting a social component within the elicitation of emotion. Choi et al. discovered inconsistencies regarding the relationship between categorical emotions and dimensional emotions, which may reflect cultural differences (Choi et al., 2015), whereas Jeon et al. (2018) attributed differences in reported emotions to different connotative meanings and semantics rather than the emotions themselves. It seems likely that, the universal character of emotion applies to the human set of emotions, whereas a cultural impact takes place more on the emotion regulation stage (cf. Mesquita and Frijda, 1992).

Moreover, fundamental differences between studies lie in their instructive process – that is to say, whether the participants were requested to report “*how the sound makes you feel*” (Cain et al., 2013) vs. “*how the sound environment is*” (ISO/TS 12913-2). Accordingly, Axelsson (2011) defined affective quality as a property of the stimulus that refers to its capacity to change our emotional responses thereby capturing the notion of *perceived* emotions. Kallinen and Ravaja (2006) observed in the context of music-induced emotions that, even though the *perceived* and *felt emotions* were more or less the same, they also demonstrated differences. Thus, it is likely that differences in the determination of emotion dimensions are due to the missing distinction of *perceived* emotions (*assigned* intrinsic property of the stimulus) and *felt* emotions (*elicited* emotions within the individual).

Overall, it appears that the first two dimensions discussed, *calmness/pleasantness* and *activity/eventfulness*, frequently emerge in numerous investigations, as indicated in Table 1. These could be regarded as a preliminary standard model for the perceptual dimensions of soundscapes (cf. Davies et al., 2014). Nevertheless, the search for additional dimensions to complement the widely established standard model appears to be ongoing, as current studies are still producing results that cannot yet be generalized across all contexts.

## What Determines Emotional Responses to Acoustic Environments?

In daily life, the various types of external stimuli that humans receive across different modalities have powerful effects on evoked emotions, influencing decision-making and subsequent behavior (Yang et al., 2018). However, the link between external stimuli and elicited emotions is still subject to extensive research. If the intrinsic properties of soundscapes leading to certain basic emotions are well understood, soundscape designers could intentionally create emotional soundscape compositions to evoke a target mood (Fan et al., 2016).

Axelsson (2011) highlighted the importance of *information load*, which drives one’s affective responses to stimuli. Aesthetic appreciation is grounded in the relationship between the amount of information of stimuli and people’s capacity to process this information, which leads to emotional responses. According to Axelsson (2011), the amount of information of a stimulus is absolute while the degree of information load is relative, depending on the individual’s processing capacity. In this approach it appears obvious that emotional responses are not solely dependent on the stimulus but are also a part of the perceiving individual. The notion of information load agrees with findings of Mehrabian and Russell, who used the concept of information rate related to meaningfulness, familiar events versus novel, and unexpected, surprising events (Mehrabian and Russell, 1974).

van den Bosch et al. (2018) related affective qualities to the indicators *affordance* and *complexity* and thereby advanced the establishment of *audible safety* as a driving force of appraisal. Affordance can be understood as cues from the environment that immediately allow the detection of function, and these cues in turn furnish behavior (Gibson, 1979). Using an evolutionary perspective, *audible safety* is an important cue in environments for warning humans of potential danger. Auditory environments that lack considerable *audible safety* require people to become vigilant and alert, resulting in stress and appraised unpleasantness. This perspective leads to the assumption that observed affective quality dimensions reflect old evolutionary motives of surviving in, coping with, and flourishing in an environment. The concept of *audible safety* resembles the semantic dimension of *control/power* observed by Gehm and Scherer (1988). Human beings appraise their soundscapes based on the level of safety they attribute to them, which guide emotional response and behavior (van den Bosch, 2015). This notion implies that soundscapes are not only appraised through emotional-based factors, but also by the extent of safety attributed to them. van den Bosch (2015) argued that the understanding of the acoustical properties of a place is far less important than understanding how that place influences a person emotionally. In the context of pictures as affective stimuli, (Bradley and Lang, 2007b) claimed that no obvious physical parameters can be used to organize emotional stimuli and to predict emotion. As the general concept of psychophysics postulates a measurable relationship between physical stimuli and the perceptions they produce, the search for the causes of emotion laying outside the human mind appears consequential. According to Frijda (1986), the links between stimulus and response are prewired, innate stimulus-response connections.

When it comes to sound stimuli, studies have shown that the affective quality of sounds, including acute physiological reactions, do not depend solely on the intensity of sounds (Bradley and Lang, 2000). This observation is fully in line with the soundscape theory, which assumes that soundscape exists through perception of the acoustic environment influenced by a multitude of factors (International Organization for Standardization, 2014). Accordingly, Davies et al. (2013) pointed to physiological experiments demonstrating that the body and brain respond to emotional content as well as simple noise levels. Bradley and Lang (2007a) reported that about 14% of the arousal variance concerning the set of International Affective Digitized Sounds IADS could be attributed to sound intensity variations. The IADS database consists of 167 natural sounds of 6 s duration that are common in daily life, which elicit different responses on the affective dimensions of *valence*, *arousal*, and *dominance* (Choi et al., 2015). Yang et al. (2018) confirmed the findings of Bradley and Lang and observed that the relationship between a physical intensity of sound and *valence* looked more complex and that classical level indicators explained only a few percentage points of the total variance.

**FIGURE 3:**
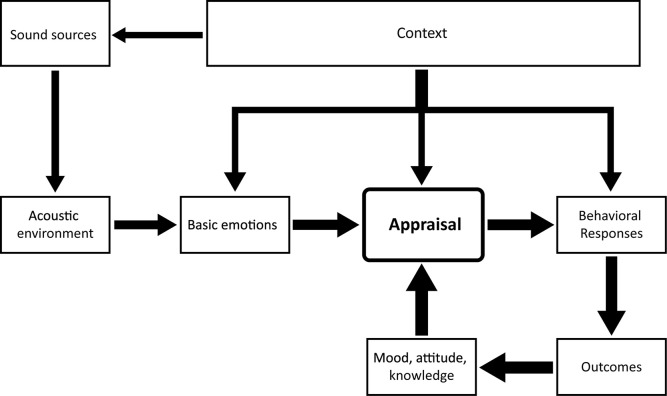
Conceptual framework of the emotional construct of soundscape [adapted from ISO 12913-1 (International Organization for Standardization, 2014)].

Figure 3 proposes a conceptual framework for understanding the process of emotional responses triggered by a soundscape, drawing from the various outcomes of previous research on emotions induced by acoustic environments. The diagram builds directly on the conceptual framework for a soundscape laid out in ISO 12913-1, which describes the process of perceiving an acoustic environment in context (International Organization for Standardization, 2014). The factor *context* continues to stand for the interactions between an individual and their (*acoustic*) *environment* (sound sources and their specific configuration), including all interrelationships in space and time between person, activity and place (International Organization for Standardization, 2014). Context here also includes elements such as the personal history, life experiences, and cultural background of the individual. The new conceptual framework introduced above, which squarely integrates facets of emotion in its structure and organization, stands apart from the known framework in the feedback loop anchored by appraisal. Here, the initial affective appraisal of a soundscape influences first short-term behavioral responses (such as moving away from the area), which in turn influence longer-term outcomes (such as habits or health effects). The resulting shifts in mood, attitude, and knowledge held by an individual may then modify prospective appraisals, leading to modified responses and so on. The conceptual framework emphasizes the importance of the frequently unconsciously elicited (basic) emotions by a soundscape, which exert influence on individuals’ behavior, well-being, and health without one being aware of it.

It appears that most soundscape research dealing with emotions does not differentiate basic emotions and appraisal. It is necessary to understand the nature of emotion and emotion processing as they are increasingly applied in studies that map soundscape (emotion) descriptors to physical indicators. The challenge is that most indicators do not consider context and meaning. Research has shown that emotional responses to sounds allegedly devoid of meaning seem to imply physical characteristics that induce affect (Västfjäll, 2012). But without considering the meaning attributed to sounds and situations, a process that always occurs in the real perceptual world, acoustical indicators do not allow comprehensive prediction of the (emotional) responses. Accordingly, van den Bosch (2015) explained that the acoustical properties of a place are far less important than understanding how the direct experience of that place influences a person emotionally. The pursuit of identifying the determinants of emotions beyond physical indicators appears justified.

It appears that the underlying mechanism to explain *pleasure* and *arousal* is related to the degree of *order* and *variation*, and these terms point the way for identifying appropriate (acoustic) indicators. The different endeavors to determine valid indicators with large amounts of explained variance illustrate that non-acoustic indicators must be considered. Västfjäll et al. (2003a) asserted that multimodal affective perception of an environment differs from unimodal perception. Consequently soundscape, as a multi-dimensional perception of an (acoustic) environment, requires the consideration of multimodal affective perception. This indicates the necessity in predictive models to integrate different sensory modalities. However, emotional reactions to short sound situations observed in experiments, which represent only brief glimpses, cannot simply be attributed to the operation of different underlying ‘motivational states’ in real life (Hall et al., 2013).

It seems that only one conclusion can be drawn from the hunt for the underlying indicators so far: before it is possible to establish predictive models of soundscape, it is necessary to fully agree upon the necessary descriptors to be predicted (Aletta et al., 2016). Although there is a growing body of knowledge regarding the predictability of emotion-related soundscape descriptors by means of acoustic and non-acoustic indicators, the comprehensive mixture of models, equations, and formulas using a wide variety of different indicators shows the general lack of consensus between researchers regarding the roots and causes of soundscape emotion and appraisal.

## Conclusion

In the 1970s, the soundscape pioneer Schafer demanded that the soundscape analyst must begin by discovering the significant features of the soundscape (Schafer, 1977). According to the latest soundscape research, elicited emotions are significant soundscape features aside the component sounds themselves. The explicit incorporation of emotions into soundscape research appears to be highly justified. Emotions elicited by soundscapes do not merely affect how we experience the sounds around us; they also color other information we process, such as the interpretations of people and events (cf. Ma and Thompson, 2015). It seems that emotion is a simple reaction to a soundscape as well as a fleeting source for several major, less evanescent phenomena. However, the exact role of emotion in the context of soundscape has not yet been clarified.

Emotion and affect can be measured in terms of physiological (re)activity, (overt) behavior, and affective self-reports. So far, soundscape research has turned its attention mostly to the measurement of verbal reporting on emotions and affect (Kuppens et al., 2012). However, it seems that a methodological distinction is rarely made between requesting related reports to intrinsically or extrinsically triggered emotions. Although the impact of the missing distinction between empirical outcomes might be minor, it may be possible that a stimulus can elicit a felt emotion differently than the emotional quality perceived by the listener (Kallinen and Ravaja, 2006; Zhang and Kang, 2020).

The inclusion of emotion-related elements into the common conceptual framework of ISO/TS 12913-1 opens the door to a progressive integration of emotion theory within soundscape and promises to guide future research substantially. In contrast to the ISO framework, the modified conceptual framework introduced in this article includes the loop of solidified emotions transformed into mood and attitudes entering future appraisals. The distinction between the different stages of emotion and appraisal including long-term effects must guide further research.

By currently accepting *hedonic valence* (pleasantness) and *arousal* or *activation* (eventfulness) as the main affective descriptors of soundscape appraisal among soundscape researchers (Davies et al., 2014), the field of soundscape study has initiated the hunt for underlying indicators (van den Bosch et al., 2018). It seems that those descriptors of soundscape appraisal can be substituted with common descriptors such as *annoyance* or *quality* (Aletta et al., 2016). The *Pleasure* and *arousal* dimensions that underlie affective judgments represent appetitive and defensive motivation, leading to responses and outcomes as described in the ISO 12913-1 (International Organization for Standardization, 2014). According to Kang et al. (2016), the commonly identified dimensions put emphasis on emotion linked to the appraisal of soundscapes and therefore need to be addressed in soundscape research. However, the emotion-stimulating potential of acoustic environments on human beings is still not comprehensively understood. “*We often experience emotions as happening to us, not as chosen by us. We do not simply decide when to have a particular emotion*” (Ekman, 1994). Therefore, a better understanding of emotions’ causes and effects is essential for any design of soundscapes. Unfortunately, emotions promoted by vibrant and lively soundscapes such as those in public urban areas still lack deeper investigations that incorporate emotion theory (Carvalho et al., 2019). However, studies have shown that emotional responses to soundscapes largely resemble emotions otherwise induced by the other senses (cf. Axelsson, 2011). This feeds the hope of developing a universal concept referring to the link between stimulus and elicited emotion independent of the sensory domain. More research will be necessary to determine possible interactions between various sensory responses to emotion.

The recent progress made within soundscape research of establishing emotion-related categories and dimensions as a core principle in soundscape research offers new options in characterizing acoustic environments from the perspective of perception. It marks significant advancement compared to the simplified, singular focus on annoyance from noise research that preceded soundscape inquiry. Beyond the almost established dimensions, it seems necessary to continue work on context-related descriptors like *affordance, coherence*, or *congruence*. Supported by emotions, perception always encompasses the conversion from sensory input to something coherent and meaningful. These categories are particularly important because pleasantness is not the only design motif employed in creating preferred soundscapes. As Davies et al. (2014) observed, participants designed soundscapes based on what was expected or appropriate rather than simply on what they liked. However, aspects like expectation or appropriateness involve cognitive processing and go beyond automatic emotions elicited by the very moment.

Research on emotion in soundscape opens exciting new research pathways. By understanding the emotional responses in different soundscapes, the knowledge of the acoustic environment might help to approach the management of urban sound as a resource for design practice (Carvalho et al., 2019).

## Future Research Tasks

It is beyond doubt that a deeper understanding of emotions elicited by soundscapes and their measurability would be a significant step forward for soundscape research. It would allow for improving perception-related assessment of actual soundscapes as well as promoting advanced design techniques. However, significant questions remain:

(1) What are the limits on the reportability of emotion experience? Can we exclusively rely on self-reported emotional experiences, assuming that the most important affective qualities are accessible by consciousness? To what extent do studies reveal information about the nature of emotion and not only about the nature of semantic concepts underlying the used attributes and scales? (Russell, 1980; Gehm and Scherer, 1988; Nielsen and Kaszniak, 2007).
(2) To what extent does the very act of reporting alter the emotional response itself? (Nielsen and Kaszniak, 2007; Rottenberg et al., 2007).
(3) How is emotion temporally structured? What is the time window for measuring experiential, behavior and physiological responses? In what way are long-lasting emotional states composed of single fleeting, evanescent emotions? Do human beings use heuristics when reporting their emotions over short episodic versus longer time frames? What is the relationship between retrospective measures and aggregated instantaneous measures? (Feldmann Barrett, 1997; Robinson and Clore, 2002; Nielsen and Kaszniak, 2007; Rottenberg et al., 2007; Gärling et al., 2020).
(4) What sorts of reporting schemes are best suited to the different emotion dimensions and affective qualities, and are these schemes culturally invariant? (Mesquita and Frijda, 1992; Nielsen and Kaszniak, 2007).
(5) As human emotion is relational and individual, is it worthwhile separating the intrinsic emotional potential of the environment from the different *appraisal histories people have* and different *affect intensities as an individual magnitude of emotional responsiveness* which influence emotions? (Larsen and Diener, 1987; Lazarus and Smith, 1988).

It seems that the lively hunt for underlying indicators of the established fundamental dimensions of emotion might obstruct the necessary view on fundamental but still unanswered theoretical issues. The measurement of emotion for soundscape studies is only of additional value if researchers work on the fundamental theoretical questions before driving headlong into more field-based research initiatives.

## Author Contributions

All authors listed have made a substantial, direct and intellectual contribution to the work, and approved it for publication.

## Conflict of Interest

The authors declare that the research was conducted in the absence of any commercial or financial relationships that could be construed as a potential conflict of interest.

## Footnotes

1 *Affect* and *emotion* are often used interchangeably in literature, although these terms intend to denote different phenomena. The majority of theories considers emotion an integral part of the superordinate category of affect (Gross, 2010). Affective phenomena thus go beyond emotion and incorporate further aspects like personality traits or well-being. In the following text, there is no sharp distinction made between these terms – as no uniformly accepted distinction is available, both aspects are highly connected and of utmost importance to soundscape perception.
